# The syntrophic network of pyrene degradation by the synthetic halophilic consortium PES

**DOI:** 10.3389/fmicb.2026.1899654

**Published:** 2026-08-14

**Authors:** Yilong Wen, Haoze Lu, Lin Wang, Han Zhao, Guang Guo, Fang Tian, Chongyang Wang, Shuxian Dang

**Affiliations:** 1 Miami College, Henan University, Kaifeng, Henan, China; 2 College of Environmental Engineering, Nanjing Institute of Technology, Nanjing, China

**Keywords:** pyrene, synthetic consortium, 4, 5-dicarboxyphenanthrene, bioemulsifier, syntrophic network

## Abstract

Owing to the high osmotic pressure, the degradation of high-molecular-weight polycyclic aromatic hydrocarbons (HMW-PAHs) in saline environments is quite difficult, leading to a scarcity of research on halophilic PAH-degrading bacteria. In this study, a halophilic synthetic consortium, PES, comprising *Pelagerythrobacter* sp. N7 and *Salinicola* sp. A11, was constructed on the basis of the bottom-up strategy. The syntrophic network for pyrene degradation by the consortium PES was investigated on the basis of genomic sequencing, functional gene annotation and expression, intermediates detection and bioemulsifier synthesis analysis. Consortium PES completely degraded 50 mg/L pyrene at 5% salinity in 10 days. Neither of strain N7 or strain A11 was identified able to complete degrade pyrene alone. Among which, strain N7 was responsible primarily for the upstream degradation of pyrene with a slow degradation rate and a low degradation efficiency. Intermediate 4,5-dicarboxyphenanthrene was detected highly accumulated in the pyrene degradation process by strain N7. Strain A11 exhibits no direct pyrene-degrading capability. Combined with the results from bioinformatic analysis and RT-PCR, strain A11 was proposed to supplies a candidate 4,5-dicarboxyphenanthrene decarboxylase to cooperate with strain N7 for complete pyrene degradation. Meanwhile, strain A11 was predicted able to produce bioemulsifier, further improving the pyrene degradation efficiency of PES consortia. Genes encoding gentisate 1,2-dioxygenase in strain N7, catechol 1,2-dioxygenase in strain N7 and A11 were detected highly expressed in pyrene degradation process. To our knowledge, this is the first study in which a syntrophic network of HMW-PAH degradation by a halophilic synthetic consortium was proposed, and 4,5-dicarboxyphenanthrene was identified as the key intermediate during pyrene syntrophic degradation. The halophilic synthetic consortium, PES, exhibited good environmental tolerance, providing an important theoretical basis and valuable microbial resources for the removal of PAHs from saline environments.

## 1 Introduction

Polycyclic aromatic hydrocarbons (PAHs) are a group of significant persistent organic pollutants. PAHs containing four or more rings are classified as high-molecular-weight polycyclic aromatic hydrocarbons (HMW-PAHs) (Beygisangchin et al., 2026). PAHs accumulate in saline and hypersaline environments, including estuaries, coastal soils, marine sediments, and intertidal zones (Dai et al., 2022; Lv et al., 2020). The biodegradability of PAHs significantly decreases with increased salinity (Li et al., 2022), which in turn enables PAHs to pose more pronounced environmental hazards in saline environments (Naidoo and Naidoo, 2021). Therefore, the degradation of PAHs in saline environments has attracted widespread attention in recent years.

As a representative HMW-PAH, pyrene (C_16_H_10_) is known for its relatively strong hydrophobicity, biotoxicity, and resistance to natural degradation by photolysis or chemical oxidation. Currently, several single strains that utilize pyrene as their sole carbon source have been isolated. Typical examples of these conventional degraders include *Mycobacterium* sp. PYR-1, *Brevibacterium* sp. MK9, and *Bacillus* sp. B3 (Karmakar et al., 2024; Kim et al., 2007; Sun et al., 2025). Highly efficient halophilic degraders remain exceptionally rare. Among the few documented cases, the halophilic bacterium *Vreelandella* sp. DM1 achieved a 58% degradation rate within 15 days (Duraimurugan et al., 2025), while the salt-tolerant marine strain *Rhodococcus* sp. MSA14 degraded 30% of 0.2 g/L pyrene over 27 days (Embarcadero-Jiménez et al., 2024). Research on pyrene-degrading halophiles is still limited. In-depth investigations on pyrene degradation by halophiles are needed to elucidate the mechanisms underlying HMW-PAH degradation in saline environments.

The degradation of PAHs by several synthetic consortia have been reported. The construction of PAH-degrading synthetic consortia using a bottom-up approach enables superior mechanistic clarity and application control, and these consortia were constructed by combining pure isolates on the basis of their whole-genome information. The consortium TMC composed of *Bacillus* sp. HG01, *Pseudomonas* spp., and *Pseudomonas* spp. constructed by Ghorbannezhad et al. (Ghorbannezhad et al., 2022) was reported to be able to degrade 79% of pyrene in 14 days. Moreover, a rationally designed consortium of five cultivable bacteria isolated from mangrove sediment, which were selected for their genomic roles in initial oxidation, intermediate metabolism, and biosurfactant production, reduced the degradation time for 100 mg/L pyrene from 6 days to 3 days (76–95% removal) (Wanapaisan et al., 2018). Synthetic microbial consortia have garnered widespread interest because of their superior PAH degradation performance and functional integrity, yet reports on halophilic synthetic consortia targeting pyrene are still scarce. Furthermore, biosurfactants and bioemulsifier have been proven to enhance the biodegradation rate of PAHs by increasing their solubility and mass transfer rates (Dhote et al., 2018; Kumari et al., 2014). Incorporating surfactant-producing strains into synthetic microbial consortia can significantly promote the PAHs-degrading capacity of the community (Qin et al., 2022; Wanapaisan et al., 2018). Therefore, the synthesis and release of biosurfactants and bioemulsifier constitute an essential component of the microbial synergistic degradation process of PAHs.

Synthetic consortia exhibit faster degradation rates and superior environmental adaptability in the degradation of PAHs compared with pure cultures. Research on the construction and degradation mechanisms of synthetic consortia is of great theoretical significance and practical value for the remediation of environmental contaminants. In this study, a halophilic synthetic pyrene-degrading consortium, PES, was constructed by 2 halophilic pure cultures, Pelagerythrobacter sp. N7 and Salinicola sp. A11. The syntrophic degradation mechanisms of pyrene by consortium PES as saline environments was studied based on the results of genomic analysis, identification of intermediate metabolites, functional gene expression analysis, and bioemulsifier synthesis detection. To our knowledge, the PES consortium is the first synthetic pyrene-degrading consortium composed of moderate halophiles, and 4,5-dicarboxyphenanthrene was proposed as the key intermediate in the synthetic degradation process. This study provides important theoretical insights into the syntrophic degradation mechanisms of pyrene in saline environments and a significant basis for the construction of synthetic PAH-degrading consortia.

## 2 Materials and methods

### 2.1 Chemicals and reagents

Pyrene (97%), phenanthrene (98%), 4,5-dicarboxyphenanthrene (97%), 4-carboxyphenanthrene (97%), phthalic acid (analytical grade), salicylic acid (99.5%), and gentisic acid (98%), were purchased from Sinopharm Chemical Reagent Co., Ltd. (Shanghai, China). Organic solvents, including dichloromethane and acetone, were of chromatographic grade and were also purchased from Sinopharm Chemical Reagent Co., Ltd. (Shanghai, China). The Fast DNA Spin Kit for Soil used for genomic DNA extraction was provided by MP Biomedicals Co., Ltd. (American).

### 2.2 Isolation and culture of halophilic bacterial strains

Strains A11 and N7 were isolated from the PAH-degrading consortium 5H (Fan et al., 2022), which was enriched from mixed saline soil samples perennially polluted with PAHs in Shanxi Province, China. In this study, 20 mL consortium 5H cell suspension was added into 180 mL fresh modified clean sea salt-defined media (SSDM) containing (per liter) NaCl (39.72 g), MgCl_2_⋅6H_2_O (3.45 g), MgSO_4_⋅7H_2_O (5.22 g), CaCl_2_ (0.75 g), (NH_4_)_2_SO_4_ (2.0 g), K_2_HPO_4_ (0.5 g), KCl (2.1 g), NaHCO_3_ (0.1 g) and 1 mL/L vitamin solution and trace element solution, with 100 mg/L phenanthrene as the sole carbon source (Zhao et al., 2009). After incubation at 30 °C for 72 h with shaking at 120 rpm, 100 μL of consortium 5H suspension was spread onto SSDM agar plates supplemented with 100 mg/L yeast extract (SSDM-Y) for the isolation of other strains, and another 100 μL cell suspension was spread onto solid SSDM containing 10 mg phenanthrene (SSDM-P) for the isolation of PAH-degrading strain (Li et al., 2024). All plates were incubated in darkness at 30°C for the growth of colonies. Strain N7 was selected from SSDM-P plate with clear degradation zone around the colony. Strain A11 was selected from SSDM-Y plate. Both strains were cultured in liquid SSDM for cultivation at 30 °C with shaking at 120 rpm. The medium constitution was identical to that of the isolation plates, with agar omitted.

### 2.3 Whole-genome sequencing, assembly and annotation

A pure culture of strain A11 was cultivated in 20 mL of 5% SSDM supplemented with 100 mg/L yeast extract at 30 °C with shaking at 120 rpm for 4 days. After incubation, 5 mL cells suspension was collected and centrifugated at 12,000 rpm for 5 min at 4 °C for DNA extraction. The genomic DNA was extracted from cell pellets using the Fast DNA Spin Kit for Soil (MP Biomedicals Co., Ltd., USA) following the manufacturer’s instructions. Whole-genome shotgun sequencing was performed by GENEWIZ Co. (Hangzhou, China) using a paired-end 150-bp strategy on the Illumina sequencing platform (Illumina, San Diego, CA, USA). Raw reads were quality-filtered using fastp (version 0.23.0) by removing adapter sequences, reads containing more than 14 ambiguous bases, reads in which fewer than 40% of bases had Phred quality scores of at least 20, and reads shorter than 75 bp. The quality-filtered reads were assembled using Velvet (version 1.2.10), with assembly parameters optimized by comparing different k-mer values, and Unicycler (version 0.5.1), with the Unicycler output used as the draft genome assembly for subsequent analyses. Assembly statistics were evaluated using QUAST (version 5.3.0), and genome completeness and contamination were assessed using CheckM2 (version 1.1.0). The quality-filtered reads were mapped to the draft genome using Bowtie2 (version 2.5.5). The alignments were processed using SAMtools sort and flagstat (version 2.0.8), and sequencing coverage was calculated using SAMtools coverage (version 1.22).

Protein-coding genes were predicted using Prodigal (version 2.6.3), tRNA genes using tRNAscan-SE (version 1.3.1), rRNA genes using Barrnap, and other ncRNAs using cmscan (version 1.1.2) against the Rfam database (version 12.0). The functions of the predicted protein-coding genes were annotated using multiple databases, including the Kyoto Encyclopedia of Genes and Genomes (KEGG), Gene Ontology (GO), Clusters of Orthologous Groups (COG), and Carbohydrate-active Enzymes (CAZy). To determine the phylogenetic position of strain A11, its 16S rRNA gene sequence was extracted from the genome and compared with sequences in the NCBI database using the BLAST program. A phylogenetic tree was subsequently constructed using the neighbor-joining (NJ) method with 1000 bootstrap replicates in MEGA 12.0 software, as described previously (Wang et al., 2017). The sequencing data for strain A11 submitted to NCBI database (accession number: SRR38848454).

### 2.4 Construction of a synthetic consortium for pyrene degradation

After cultivation, cells of strain N7 and strain A11 pure cultures was collected by centrifugation, respectively. The collected cells were further washed and resuspended by fresh 5%-SSDM to OD600 value of 0.2. The 2 cell resuspensions were mixed at 1:1 volume ratio to construct the synthetic consortium PES (Wang et al., 2020). Biodegradation assays of consortium PES were conducted in 200 mL of 5% salinity SSDM with 50 mg/L pyrene as the sole carbon source, with initial OD600 value of 0.015. Abiotic controls containing only SSDM and pyrene were also included to assess abiotic loss. Three independent flasks were prepared for each treatment and incubated at 30°C with shaking at 120 rpm in darkness. Strain N7 and the synthetic consortium PES were incubated for 14 and 10 days, respectively.

### 2.5 Measurement of the residual pyrene concentration

To quantify residual pyrene, 3 mL samples from the strain N7 monoculture and the synthetic consortium PES were collected once per day and transferred into glass extraction tubes fitted with PTFE-lined screw caps. Each tube was placed on a rotary extractor for 1.5 h (Li et al., 2024; Wang et al., 2020), after which the dichloromethane extracts were filtered through 0.22-μm organic-phase-compatible membrane filters into HPLC vials. Residual pyrene and the accumulated 4,5-dicarboxyphenanthrene were quantified using a high-performance liquid chromatography system (HPLC; Shimadzu, Japan) equipped with an XB-C18 reversed-phase column (5 μm, 4.6 × 200 mm). The mobile phase consisted of methanol and ultrapure water (90:10, v/v) at a total flow rate of 1.0 mL/min. Detection was performed at 254 nm, the column temperature was maintained at 40°C, and the analysis time was 20 min (Wang et al., 2020). Standard substance of pyrene and 4,5-dicarboxyphenanthrene were dissolved in dichloromethane and diluted for determine the retention time and the construction of working curves. The fresh 5% SSDM with 50 mg/L pyrene and placed at same condition was set as a control.

### 2.6 Degradation of pyrene by the synthetic consortium under different salinities and pH values

The adaptability of the synthetic consortium to environmental factors was evaluated by monitoring pyrene degradation under various salinity levels (1%, 3%, 5%, 8%, and 10%) and pH values (5.0, 6.0, 7.0, 8.0, 9.0, and 10.0). The salinity gradients were prepared by proportionally adjusting the concentrations of NaCl, MgCl_2_⋅6H_2_O, and MgSO_4_⋅7H_2_O while keeping the other SSDM components unchanged (Wang et al., 2018). The respective concentrations of NaCl, MgCl_2_⋅6H_2_O, and MgSO_4_⋅7H_2_O were 7.94, 0.69, and 1.04 g/L at 1% salinity; 23.83, 2.07, and 3.13 g/L at 3% salinity; 39.72, 3.45, and 5.22 g/L at 5% salinity; 63.55, 5.52, and 8.35 g/L at 8% salinity; and 79.44, 6.90, and 10.44 g/L at 10% salinity, respectively. The initial pH of the medium was adjusted using 0.5 mol/L NaOH or 1:5 (v/v) HCl solution, which was prepared by mixing HCl and water at a volume ratio of 1:5.

All the experimental groups were cultivated in 200 mL of SSDM supplemented with 50 mg/L pyrene at 30°C with shaking at 120 rpm. For the salinity experiments, samples were collected every 2 days over a 14-day incubation period to determine the residual pyrene concentration. For the pH experiments, the degradation efficiency, calculated as the percentage of pyrene removed relative to the initial concentration, was determined after 3, 7, and 10 days of incubation. The residual pyrene concentration was quantified using HPLC as described above. Abiotic controls containing only SSDM and pyrene were included for each condition. All the treatments were performed in triplicate.

### 2.7 Identification of metabolic intermediates during pyrene degradation

Metabolic intermediates were identified during pyrene degradation by the monoculture of strain N7 and the synthetic consortium. Cell suspensions were collected at the early phase and late phase of degradation, respectively. For the degradation of pyrene by synthetic consortium, 50 mL of cell suspensions was collected after incubation for 3 and 8 days. For the degradation of pyrene by strain N7 pure culture, cell suspensions were collected after incubation for 5 and 10 days. The samples from different time points were mixed in each treatment. Prior to extraction, the cells in the suspension were disrupted using a high-pressure homogenizer (JN-Mini, JNBIO Co., Ltd., Guangzhou, China) to release intracellular metabolites. The mixture was then extracted using 100 mL of dichloromethane at adjusted pH values (2.0, 7.0, and 12.0) to ensure the comprehensive recovery of acidic, neutral, and basic metabolites. After extraction, the organic phases were combined, dehydrated with anhydrous sodium sulfate, concentrated using a rotary evaporator, and further dried under nitrogen.

The dried residue was redissolved in 1 mL of dichloromethane. Derivatization was performed using N,O-bis(trimethylsilyl)trifluoroacetamide (BSTFA) containing 1% trimethylchlorosilane (TMCS) (volume ratio 99:1) to replace active hydrogen atoms with trimethylsilyl (TMS) groups. The reaction was conducted in a sealed container at 60°C for 40 min. After cooling to room temperature, the mixture was filtered through an organic membrane filter. The derivatized samples were analyzed using gas chromatography–mass spectrometry (GC–MS, Agilent 7890A GC, Inert MSD with Triple-Axis Detector, Agilent, USA) equipped with an HP-5 capillary column (30 m × 0.25 mm internal diameter × 0.25 μm film thickness). Helium was used as the carrier gas at a flow rate of 1.0 mL/min. The injector temperature was maintained at 250°C. The oven temperature was initially held at 80°C for 1 min, increased to 280°C at 5°C/min, and then held at 280°C for 10 min. Mass spectra were acquired in electron-impact ionization mode at 70 eV over a mass-to-charge ratio range of m/z 50–550. The ion source and quadrupole temperatures were maintained at 230 and 150°C, respectively. Authentic standards, including phenanthrene-4,5-dicarboxylic acid, phenanthrene-4-carboxylic acid, phthalic acid, salicylic acid, and gentisic acid standards, were processed using the same procedure for compound identification.

### 2.8 Bioinformatic analysis of candidate 4,5-dicarboxyphenanthrene decarboxylase in strain A11

To screen for candidate genes potentially encoding 4,5-dicarboxyphenanthrene decarboxylase in strain A11, decarboxylase-related protein-coding genes were initially examined on the basis of the genome annotation described above and the additional annotation results obtained using RASTtk (version 1.3.0), EggNOG (version 7.0), and BLASTP in BLAST+ (version 2.17.0). The conserved domains and putative functional sites of the candidate proteins and reference 4,5-dicarboxyphenanthrene decarboxylases were analyzed using the NCBI Conserved Domain Database (CDD) (version 3.21). The amino acid sequences of the candidate proteins and reference 4,5-dicarboxyphenanthrene decarboxylases were submitted to AlphaFold Server for three-dimensional structure prediction. The predicted protein structures were visualized using PyMOL (version 1.8) and structurally superimposed using the super command. Potential ligand-binding pockets in the candidate enzymes were identified using DoGSiteScorer on the ProteinsPlus server with the default settings.

### 2.9 Analysis of interfacial properties and bioemulsifier characterization

Consortium PES and strain N7 were separately cultivated in 5% SSDM containing 50 mg/L pyrene as the sole carbon source. Strain A11 was cultivated in 5% SSDM supplemented with 100 mg/L yeast extract. All cultures were incubated at 30°C with shaking at 120 rpm in the dark for 5 days. Four mL cell suspension was collected from each treatment. After centrifugation, washed-cell suspensions and cell-free supernatants were prepared. The washed cells were resuspended in 4 mL fresh 5% SSDM with no carbon source.

To qualitatively compare the emulsification behavior of the different test phases, the 24-h emulsification index (E24) assay was performed using soybean oil as the hydrophobic phase. For each assay, 4 mL of the test liquid phase was mixed with 6 mL of soybean oil in a sealed glass tube. The mixtures were vigorously vortexed and then allowed to stand undisturbed for 24 h at 25°C. Four mL fresh 5% SSDM were used as negative controls. Strain N7 supernatant supplemented with Tween 20 at final concentrations of 0.5% was used as an external emulsifier reference. The emulsification index was calculated as the height of the emulsified layer divided by the total height of the liquid column and multiplied by 100% (D’Almeida et al., 2024). Each treatment was prepared in triplicate.

To extract the bioemulsifier, the cell-free supernatant of the pyrene-containing fermentation broth was first collected, mixed with an excess of prechilled absolute ethanol, and incubated at 4°C to induce cross-linking and the precipitation of macromolecular flocs. The harvested precipitate was subsequently washed with cold 70% ethanol and centrifuged, and this step was repeated three times to effectively remove inorganic salts and other water-soluble impurities (D’Almeida et al., 2024). The precipitated polymer was subsequently transferred to a glass extraction tube using absolute ethanol, and dichloromethane was added for vigorous solid–liquid extraction. After the undissolved solid impurities were allowed to settle, a portion of the upper liquid enriched with the bioemulsifier was carefully pipetted. The collected extract was evaporated to complete dryness at 70°C to yield the purified solid crude bioemulsifier. Fourier transform infrared (FTIR) spectroscopy analysis was conducted using an ALPHA II spectrometer (Bruker, Germany) (Chebbi et al., 2017). The solid sample was homogeneously mixed with potassium bromide and pressed into a transparent pellet, after which the infrared absorption spectra were acquired across the wavenumber range of 4000 to 400 cm^–1^. All measurements were performed in triplicate.

### 2.10 RNA extraction and quantitative real-time PCR (RT–qPCR)

To investigate the expression of key functional genes, namely, ring-hydroxylating dioxygenase (RHD), catechol 1,2-dioxygenase (C12O), and gentisate 1,2-dioxygenase (G12O), and 4,5-dicarboxyphenanthrene decarboxylase (*hphtC-5*) during pyrene degradation, quantitative PCR was performed on the synthetic consortium cultured under 5% and 10% salinity. Fifty-milliliter cell suspensions were collected once per day from day 1 to 10 during the degradation process, and the cell pellets were harvested by centrifugation. RNA was extracted by RNAiso (Takara, Dalian) according to the manufacturer’s instructions and measured the concentration of extracted RNA using Nanodrop 2000 (ThermoFisher, Shanghai). The extracted RNA was further reverse-transcribed by a cDNA synthesis kit (ThermoFisher, Shanghai) before conservation (Wang et al., 2018).

All gene fragments were amplified in this study. The cloned DNA fragments were recovered via gel purification (Takara, Dalian), ligated with T Vector pMD20 (Takara, Dalian), and subsequently transformed into competent Escherichia coli DH5α cells. After cultured under 37°C for 48 h, plasmids were extracted from E. coli cells using the Plasmid Extraction Kit (DP103, Tiangen), and the concentration of extracted plasmids was determined with a Nanodrop. The number of plasmid copies was calculated using the formula 1. *Y* is the number of plasmids, *x* is the concentration of cDNA measured by nanodrop.


$$y=\frac{6.02\times 10^{23}\,x}{D\,N\,A\,f\,r\,a\,g\,m\,e\,n\,t\,l\,e\,n\,g\,t\,h\times 10^{9}\times 660}$$ (1)

The purified plasmids were serially diluted by 10, 10^2^, 10^3^ and 10^4^ times for the construction of standard curves for RT-PCR. Transcripts of these functional genes were quantified in an iCycleriQ (Bio-Rad, USA) using the primer pairs shown in Supplementary Table 1. Primers used were designed according to the whole-genome sequences of strains N7 and A11 by NCBI database. The RT-PCR reaction mixture contained 10 μL SYBR Premix Ex Taq™ II, 1 μL forward and reverse primers, 1 μL cDNA template, and 7 μL double distilled water (ddH2O). The primer sequences and corresponding annealing temperatures for qRT PCR are listed in Supplementary Table 1, and the amplification reaction was performed for a total of 45 cycles. Melting-curve analysis was performed from 51 to 95°C, with fluorescence acquisition after each 0.5°C increase at 10-s intervals. Each RT-PCR reaction was performed in technical triplicate. The standard curve of each functional genes was shown in Supplementary Figure 1.

## 3 Results and discussion

### 3.1 Composition and genome sequencing of the synthetic consortium PES

**FIGURE 1:**
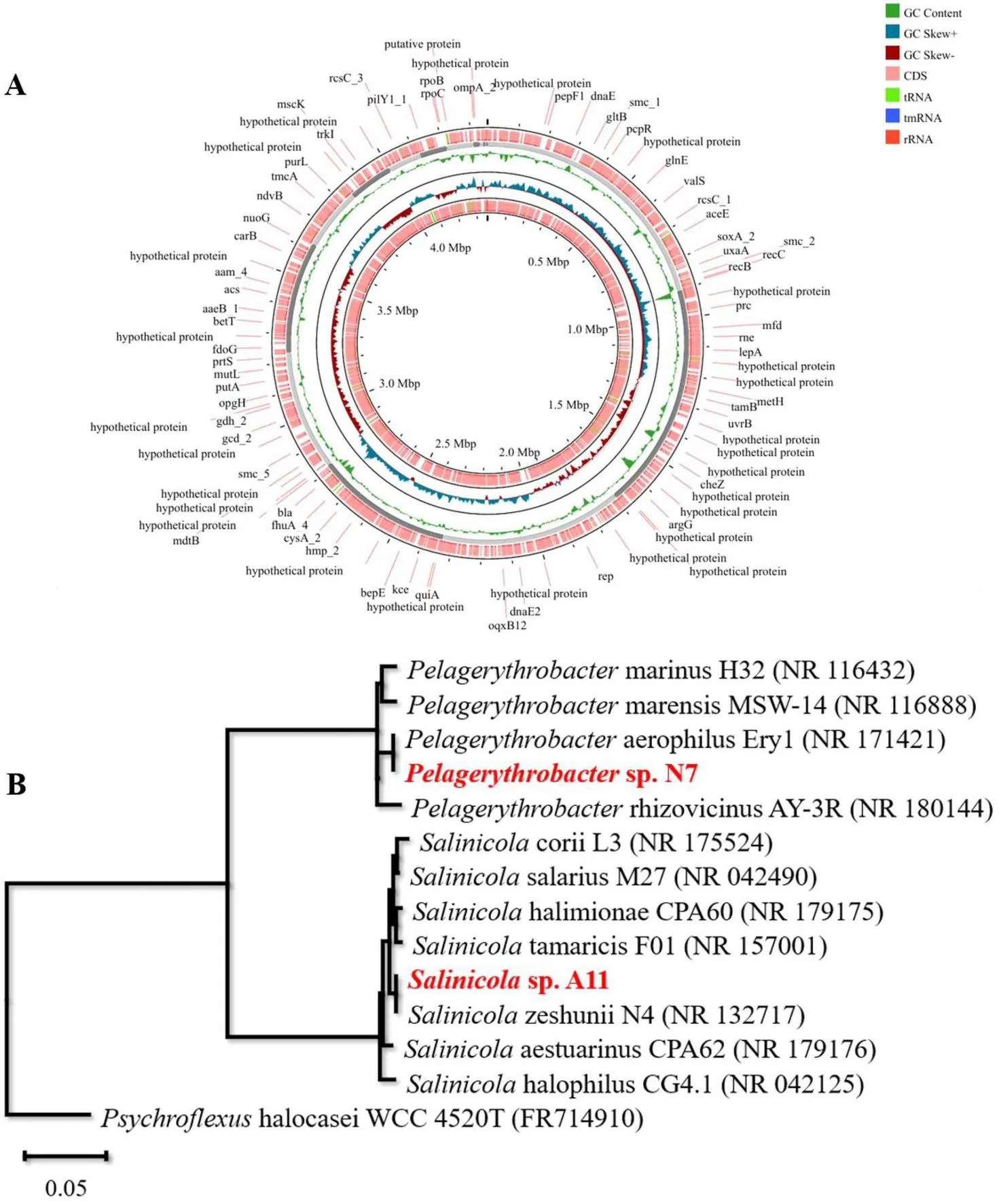
Genome map of strain A11 and strain constituents within the synthetic PES consortium. **(A)** Genomic map (4.37 Mb); **(B)** phylogenetic tree of strains N7 and A11 constructed using the neighbor-joining method with 1000 repetitions.

The synthetic consortium PES was constructed from strains N7 and A11, which were both isolated from the halophilic PAH-degrading consortium 5H (Fan et al., 2022), on the basis of the bottom-up method at a 1:1 ratio of the two strains. The isolation and genomic map of the PAH-degrading strain N7 was reported by Li et al. (2024). To provide an in-depth version of the genomic information of the consortium PES, whole-genome sequencing of strain A11 was performed. 35,246,470 clean reads were obtained after fastp filtering (5.298 Gb) with Q20 and Q30 values of 97.52% and 92.83%. CheckM2 estimated genome completeness and contamination to be 100.0% and 0.22%, respectively. When the original paired-end reads were mapped back to the final assembly, 97.85% of the reads were mapped and 95.46% were properly paired. All 4,356,676 assembly bases were covered by at least one read, corresponding to a genome coverage breadth of 100.00%, which indicating a high quality of the draft genome assembly of strain A11. The assembled whole genome of strain A11 is approximately 4.37 Mb in size, and the average GC content is 64.29%, with 18 contigs. The circular genomic map is shown in Figure 1A.

A joint phylogenetic tree (Figure 1B) constructed with representative strains using the neighbor-joining method with 1000 bootstrap replicates revealed that the core degrading strain N7 clustered within the same core branch as *Pelagerythrobacter* sp. Ery1 (NR_171421), thereby being unambiguously identified as the genus *Pelagerythrobacter* (Li et al., 2024). Strain A11 exhibited extremely high phylogenetic relatedness with *Salinicola* sp. N4 (NR_157001), forming a tight cluster on the evolutionary tree, and was formally identified as the genus *Salinicola*. On the basis of these molecular phylogenetic characteristics, the core members in the synthetic consortium PES were ultimately defined as *Pelagerythrobacter* sp. N7 and *Salinicola* sp. A11.

### 3.2 Pyrene-degrading ability and environmental tolerance of the synthetic consortium PES

**FIGURE 2:**
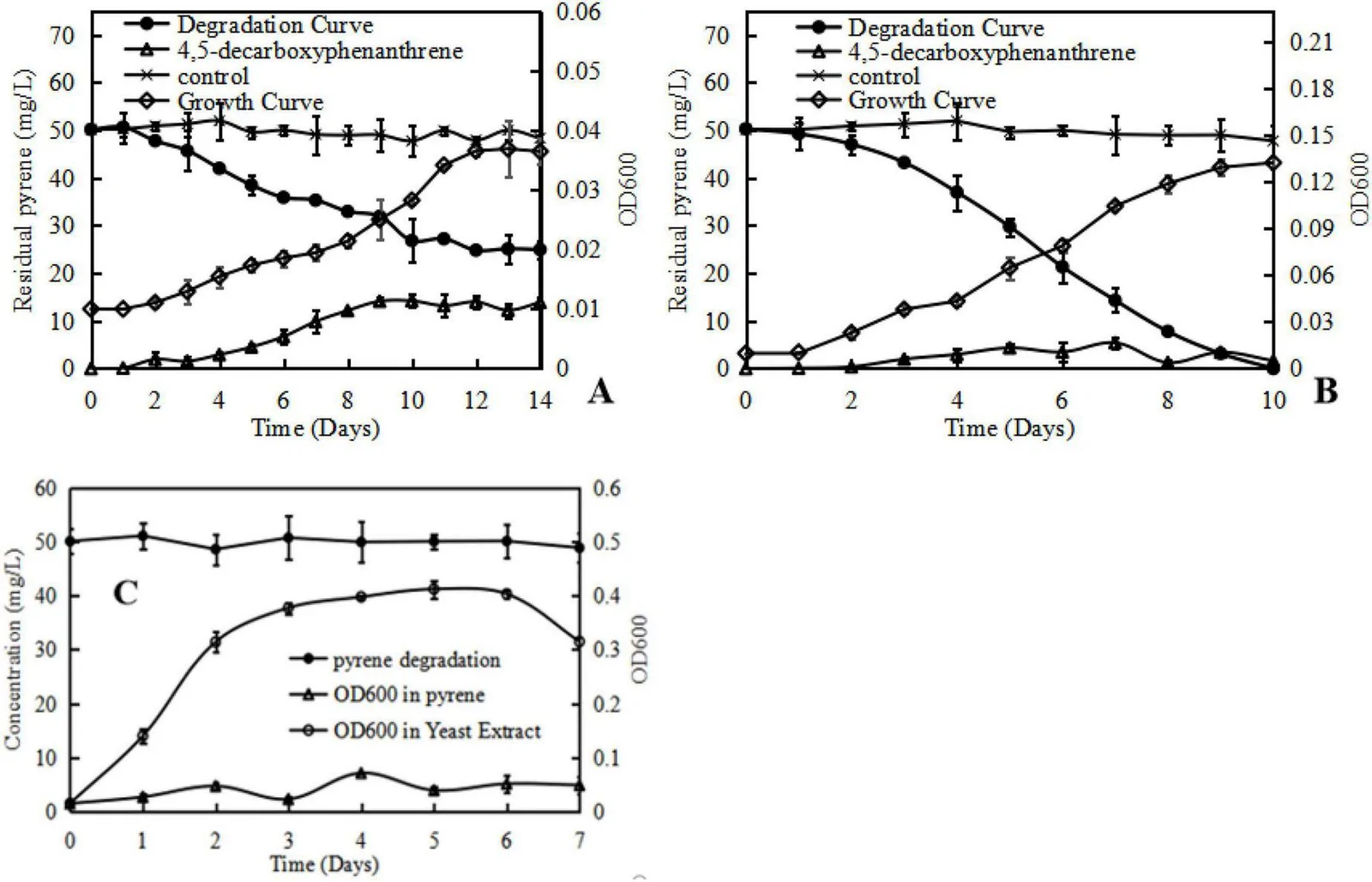
Degradation kinetics and growth curves of pyrene and accumulation of 4,5-dicarboxyphenanthrene by **(A)** strain N7 pure culture, **(B)** synthetic consortium PES and **(C)** strain A11 pure culture.

To evaluate the syntrophic pyrene degradation ability of the consortium PES, the degradation rates and growth curves of the single strain N7 and the synthetic consortium were determined in 5% salinity SSDM with 50 mg/L pyrene as the sole carbon source. As shown in Figure 2A, The ability of strain N7 to independently degrade pyrene was limited. After 14 days of cultivation, the degradation rate plateaued at 49.95%, and a low value of cell growth, indicating that strain N7 was not able to complete remove pyrene. Moreover, intermediate 4,5-dihydroxypyrene were also detected accumulated in the degradation process. After 9 days degradation, the concentration of 4,5-dicarboxyphenanthrene was riched to 12.17 mg/L, indicating the limited ability of strain N7 in the degradation of pyrene. Strain A11 was detected to be unable to degrade PAHs (Figure 2C). With the addition of strain A11, the pyrene degradation ability of the consortium PES was good. Within a 10-day cultivation period, the removal rate of 50 mg/L pyrene by the consortium PES approached 100%, with the maximum OD_600_ value reaching approximately 0.13, demonstrating a syntrophic promotion effect between strain N7 and strain A11 (Figure 2B). According to the literature, the degradation rate of the isolated halotolerant pyrene-degrading single strain *Thalassospira* sp. TSL5-1 was 41.4% for an initial pyrene concentration of 20 mg/L within 25 days under 5% salinity conditions (Zhou et al., 2016). Another degrading strain, *Rhodococcus* sp. TA13008, exhibited a degradation rate of 52–56% for 100 mg/L pyrene within 18 days with the addition of the syntrophic substrate naphthalene (Al-Thukair et al., 2020). In comparison, the synthetic consortium still retained a highly efficient pyrene degradation ability in a saline environment.

**FIGURE 3:**
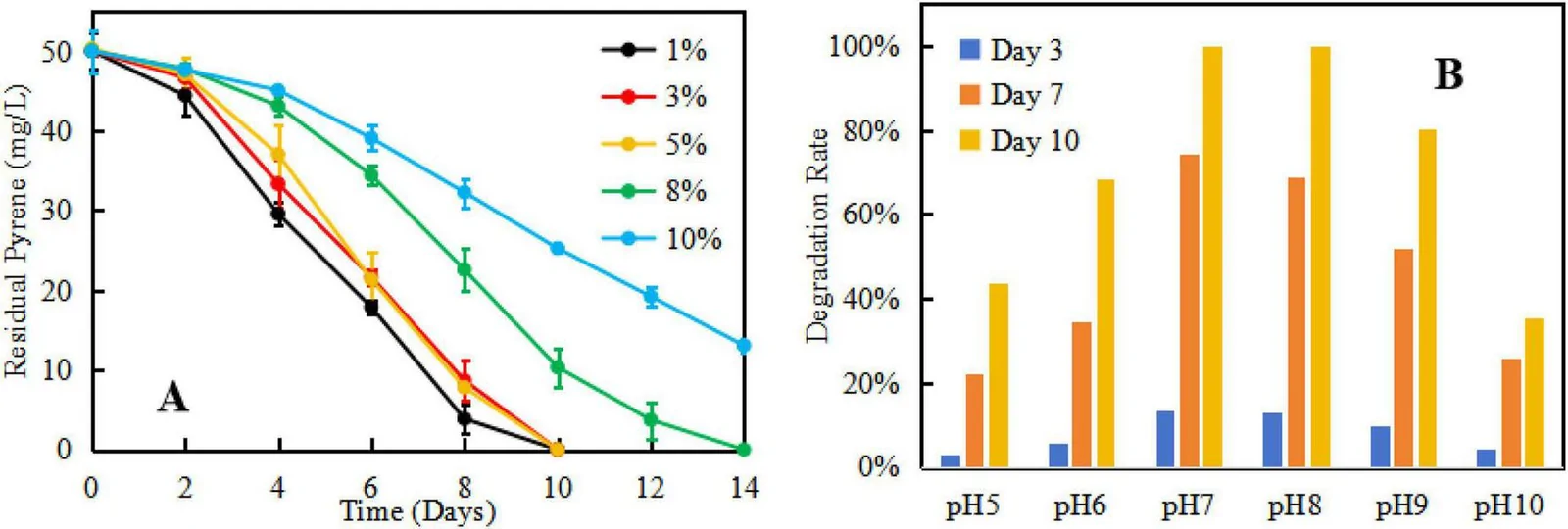
Influence of environmental factors on 50 mg/L pyrene degradation. **(A)** Degradation kinetics across salinities of 1%–10%. **(B)** Degradation rates at pH 5–10 on days 3, 7, and 10.

To evaluate the environmental tolerance of the synthetic consortium, the pyrene degradation rate of the consortium PES, was tested over a wide range of salinities (1% to 10%) and pH values (5 to 10). As shown in Figure 3A, the synthetic consortium maintained high degradation activity at salinities of 1%, 3%, and 5%, completely degrading 50 mg/L pyrene within 10 days. When the salinity increased to 8%, the degradation rate slightly decreased, yet the pyrene removal rate of the consortium was nearly 100% on day 14. Remarkably, under 10% hypersaline stress, the pyrene degradation rate still reached approximately 74% after 14 days. As reported by Nzila et al., the pyrene degrader *Achromobacter* sp. PY4 thrives in a narrow low-salinity range of 0 to 2.5%, completely losing viability once the salinity exceeds 5% or when the pH reaches 9 (Nzila et al., 2018). Moreover, although certain highly halophilic degraders can survive above 10% salinity, they fail to grow under nonsaline conditions or in acidic environments with a pH value of five or lower (Budiyanto et al., 2018). Therefore, the synthetic consortium PES showed good tolerance to changes in the salinity.

As shown in Figure 3B, the degradation efficiency approached 100% after 10 days of incubation at both pH 7 and pH 8, with the most rapid degradation occurring at pH 7. Acidic conditions at pH 5 and 6 or highly alkaline conditions at pH 9 and 10 inhibited the degradation process to varying extents. Environmental pH is another critical factor influencing microbial degradation efficiency. Related studies report that the pyrene-degrading consortium PBR performs best at pH 7.0 but loses efficiency under acidic or alkaline conditions, with the degradation rate decreasing nearly 1.8-fold under alkaline stress (Vaidya et al., 2017). As reported by Badejo et al., high salinity significantly postpones the peak expression of the dioxygenase gene *phdF*, whereas a pH of 5.5 directly suppresses key genes such as *pcaG* and *pcaH* (Badejo et al., 2013).

### 3.3 Intermediate metabolite detection and analysis of decarboxylation constraints

**FIGURE 4:**
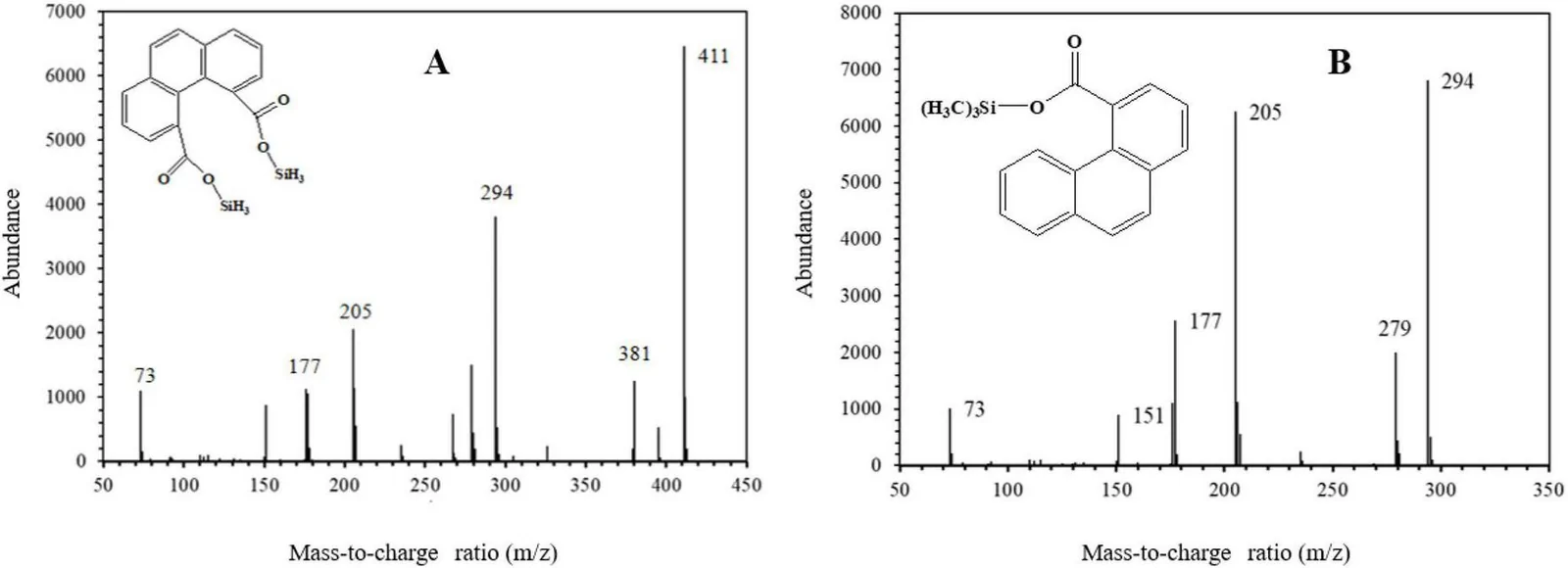
MS spectra of **(A)** 4,5-dicarboxyphenanthrene **(B)** 4-carboxyphenanthrene.

Intermediate metabolites were detected during pyrene degradation by the single strain N7 and the consortium PES at 5% salinity. The specific retention times and characteristic mass-to-charge ratios of each compound are detailed in Table 1. The metabolite detected at 40.25 min with major ions 380 (M+), 365, 290, 218, and 73 was determined to be 4,5-dihydroxypyrene. The metabolite detected at 38.10 min, with major ions at 411, 381, 294, 205, 177, and 73, was proposed to be 4,5-dicarboxyphenanthrene. The mass spectra of these two compounds are shown in Figure 4. Previous studies generally assumed that the degradation of HMW-PAHs is limited primarily by a lack of highly specific initial RHDs (Sun et al., 2024; Wang et al., 2018). However, in this study, the identification of metabolites using GC–MS revealed a distinct limiting mechanism. The RHD within strain N7 has broad substrate affinity and active site flexibility (Li et al., 2024) and successfully catalyzes the addition of two hydroxyl groups on the benzene ring of pyrene. However, in the subsequent decarboxylation step, its metabolic pathway was severely hindered. Owing to the inability to further transfer 4,5-dicarboxyphenanthrene, the metabolic pathway was disrupted, leading to the significant accumulation of 4,5-dicarboxyphenanthrene during pyrene degradation by the pure culture of strain N7.

**TABLE 1:** Identification of intermediate metabolites in pyrene degradation by strain N7 and consortium PES.

Metabolic group	Peak time (min)	Characteristic fragment mass-to-charge ratio (m/z)	Metabolite identification result
N7	40.25	380, 365, 290, 218, 147, 73	4,5-dihydroxypyrene
	38.10	411, 381, 294, 205, 177, 73	4,5-dicarboxyphenanthrene
N7 and A11	41.06	380, 365, 290, 218, 147, 73	4,5-dihydroxypyrene
	39.45	411, 381, 294, 205, 177, 73	4,5-dicarboxyphenanthrene
	37.08	294, 279, 205, 177, 151, 73	4-carboxyphenanthrene
	25.12	310, 295, 147, 73, 45	Phthalate
	21.16	267, 149, 135, 91, 74, 73, 45	Salicylic acid
	24.51	370, 311, 280, 223, 193, 128, 73	Gentisate

The impeded decarboxylation of phenanthrene-4,5-dicarboxylic acid is relatively common in microbial PAH metabolic networks. Previous studies on pyrene degradation by *Shigella* sp. PyB-6 and *Agromyces* sp. PyB-10 have also confirmed that this compound is the most abundant accumulated product (Kweon et al., 2011). An in-depth analysis from multiomics and evolutionary perspectives revealed that aromatic acid decarboxylases exhibit highly specific niche characteristics throughout the metabolic network. Decarboxylases, especially aromatic carboxylic acid decarboxylase genes, often exist in only one or two copies within the genomes of most strains (Liang et al., 2006). In the study of pyrene degradation by *Mycobacterium* sp. PYR-1, researchers have focused mostly on the study of RHDs, while decarboxylation genes such as *phtC* have remained largely ignored because of their extremely low expression levels and lack direct functional experimental verification (Qutob et al., 2022). Moreover, as the cytotoxicity and mutagenicity of 4,5-dicarboxyphenanthrene itself are relatively weak (Zhuo et al., 2025), microorganisms did not face sufficient selective pressure to evolve the decarboxylase of 4,5-dicarboxyphenanthrene.

In pyrene degradation by the consortium PES, downstream metabolites were largely detected by GC–MS analysis. In addition to 4,5-dihydroxypyrene and 4,5-dicarboxyphenanthrene, the metabolites detected at 37.08 min, 25.12 min, 21.16 min and 24.51 min were determined to be 4-carboxyphenanthrene, phthalate, salicylic acid, and gentisate, respectively, indicating the further transfer of 4,5-dicarboxyphenanthrene between strain N7 and strain A11 (Table 1). Therefore, it is reasonable to predict that Strain A11 can provide 4,5-dicarboxyphenanthrene decarboxylase to assist strain N7 in the further transformation of pyrene.

### 3.4 Proposal of 4,5-dicarboxy- phenanthrene decarboxylases in strain A11

**FIGURE 5:**
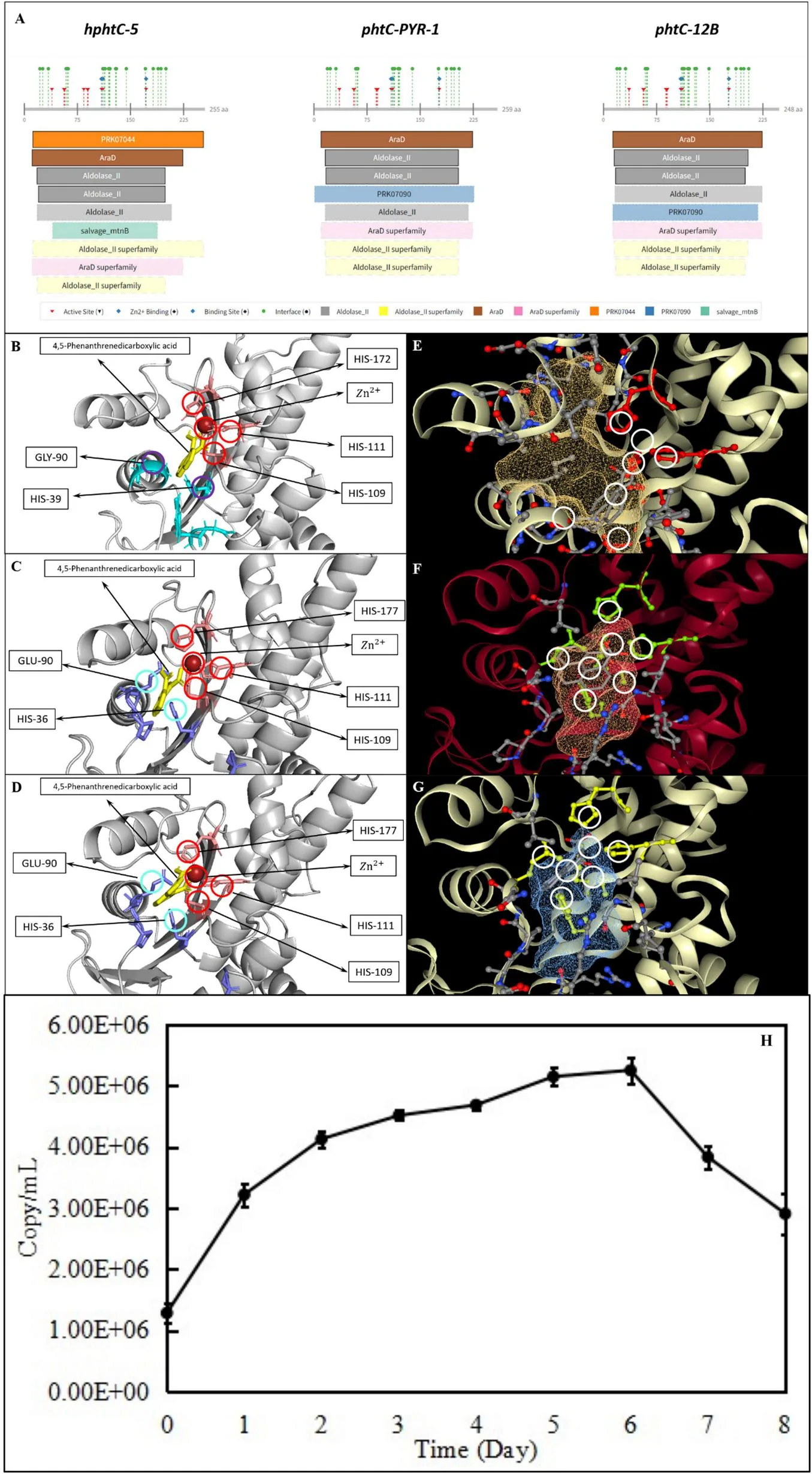
Structural and functional comparison between the key decarboxylase (*hphtC-5*) in strain A11 and reference decarboxylases (*phtC*-PYR-1 and *phtC*-12B). **(A)** One-dimensional mapping of conserved domains and key functional sites. **(B–D)** 3D structures of the catalytic centers. **(E–G)** The active site binding pockets. **(H)** The expression of *hphtC-5* in the degradation process.

Integrating genomic annotation with multidimensional computational structural biology has been proven to be an effective approach for identifying novel degrading enzymes and their substrate adaptability (Eaton, 2001). In this study, by cross-referencing multiple databases, 6 genes (*hphtC* 1-6) encoding potential decarboxylases in the genome of strain A11 were annotated. Conserved domain mapping (Figure 5A) revealed that the candidate gene *hphtC-5* is highly homologous to 2 standard aromatic compound decarboxylases in Arthrobacter sp. 12B and Mycobacterium sp. PYR-1 (Jumper et al., 2021; Kim et al., 2007; Wang et al., 2023). All 3 sequences share the same class II aldolase/adducin family conserved domains and a secondary classification of L-fuculose phosphate aldolase. Furthermore, predicted functional regions, including active sites, zinc-binding sites, and intersubunit interfaces, are highly conserved across these proteins.

To evaluate 3D structural conservation, the spatial conformation of *HphtC-5* was predicted using AlphaFold (Capitani et al., 2003). As identified by conserved domain analysis, this class of enzymes relies on a tightly configured histidine triad to coordinate the catalytic zinc ion. Microscopic active site analysis (Figures 5B–D) revealed that strain A11 formed a catalytic triad via His109, His111, and His172, with internal atomic distances of 3.01 Å, 2.85 Å, and 3.50 Å, respectively. In comparison, the internal distances of the corresponding residues in PhtC-12B were 2.96 Å, 3.06 Å, and 3.48 Å, while those in PhtC-PYR-1 were 2.99 Å, 3.18 Å, and 3.38 Å. The internal distances of the triads in all three enzymes were strictly maintained within 4 Å. Spatial superposition indicated that the coordinate offsets of the A11 catalytic triad relative to PhtC-12B were 1.30 Å (residue 109), 0.81 Å (residue 111), and 1.57 Å (residue pair 172/177), whereas the offsets relative to PhtC-PYR-1 were 1.37 Å, 0.85 Å, and 1.60 Å, respectively. These results suggested that the positional shift toward residue 172 in strain A11 does not compromise the geometric integrity or functional conservation of the metal-binding center. Additionally, the substitution of Glu90 with Gly90 likely eliminates local steric hindrance to accommodate the bulky 4,5-dicarboxyphenanthrene molecule. The subsequent shift of His36 to position 39 retains the residue type, reflecting its essential role in stabilizing hydrophobic interactions, maintaining proper folding, and regulating pH stability within the core region (Schöning-Stierand et al., 2020).

To further evaluate substrate compatibility, the geometric and physicochemical properties of the active site binding pockets were analyzed using the DoGSiteScorer (Volkamer et al., 2012) tool on the ProteinPlus (Trott and Olson, 2010) server (Figures 5E–G). The catalytic residues of PhtC-12B and PhtC-PYR-1 were restricted to secondary pockets with volumes of 222.14 Å^3^ and 242.56 Å^3^, respectively. In contrast, the catalytic residues of A11 were integrated into the primary binding pocket, which has a significantly expanded volume of 596.99 Å^3^, which is consistent with the spatial expansion predicted from the Glu90 to Gly90 substitution. With respect to the chemical microenvironment, the number of hydrophobic interactions in A11 reached 44, which was greater than the 17 and 19 observed in the reference enzymes, with the hydrophobicity ratio increasing to 0.41. Molecular docking simulations further revealed a favorable binding conformation in which the reactive carboxyl groups of the substrate are oriented toward the catalytic zinc center (Uzoigwe et al., 2015), indicating it may more suitable for catalyzing HMW-polycyclic aromatic acid. It provides the sufficient spatial capacity and hydrophobic anchoring required to stabilize bulky 4,5-dicarboxyphenanthrene, ensuring its precise alignment with the catalytic zinc center for decarboxylation. The above results indicating that despite significant sequence divergence during evolution, the core 3D folding framework and catalytic scaffold of the enzyme encoded by *hphtC-5* were highly conserved compared with those of 4,5-dicarboxyphenanthrene decarboxylase in *Arthrobacter* sp. 12B and *Mycobacterium* sp. PYR-1.

A RT-PCR test of *hphtC-5* expression during the degradation of pyrene by consortium PES was also performed. As shown in Figure 5H, The expression of *hphtc-5* reached to 5.25 × 10^6^ copy/mL in day 6. Therefore, enzyme encoded by *hphtC-5* was proposed to be the candidate decarboxylase for the decarboxylation process of 4,5-dicarboxyphenanthrene.

### 3.5 Genomic mining of bioemulsifiers and interfacial solubilization mechanisms

**FIGURE 6:**
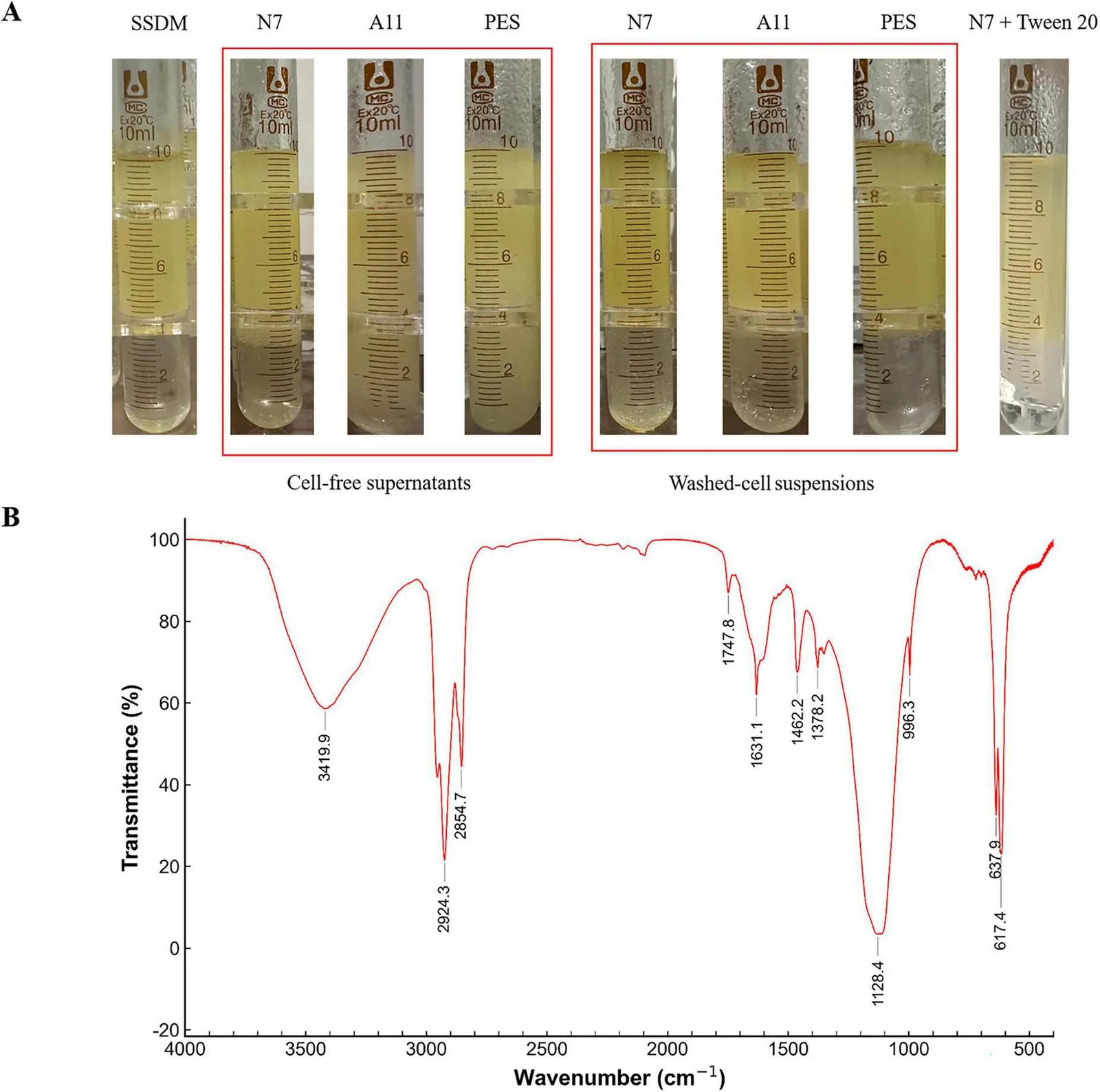
Physicochemical characterization of emulsan from strain A11. **(A)** Macroscopic emulsification phenomenon; **(B)** Fourier-transform infrared spectroscopy analysis of the extracted crude bioemulsifier.

An emulsification test verified the high efficiency and prolonged stability of the bioemulsifier synthesized by strain A11 (Figure 6A). The cell-free fermentation broth achieved a 24-h emulsification index of 68% against soybean oil. Notably, a clear aqueous phase remained at the bottom, accompanied by a distinct white flocculent polymer network at the oil–water interface. This finding indicates that such macromolecules stabilize oil-in-water emulsions primarily through strong spatial steric hindrance rather than by reducing surface tension (Chamanrokh et al., 2010). Consequently, low concentrations of this bioemulsifier can efficiently encapsulate oil droplets via spatial entrapment, leaving the bulk aqueous phase intact. The possible source of the emulsifying activity in consortium PES was further investigated by qualitatively comparing the cell-free supernatants and washed-cell suspensions of consortium PES, strain N7, and strain A11, using fresh SSDM as a negative control and N7 supernatant supplemented with Tween 20 as an external emulsifier reference. As shown in Figure 6A, Distinct and persistent emulsion layers were observed only in the cell-free supernatants of strain A11 and consortium PES, with an appearance similar to that of the Tween 20 reference. In contrast, no comparable stable emulsification was observed in the N7 supernatant, any of the washed-cell suspensions, or fresh SSDM. These observations indicate that the emulsifying activity of PES was mainly associated with extracellular components and suggest that strain A11 was the likely primary source of the extracellular bioemulsifier.

FTIR spectroscopy further investigated its chemical composition (Figure 6B). In addition to minor variations in relative peak heights, the overall spectral profile is nearly identical to that of the classic Emulsan produced by Acinetobacter sp. RAG-1 (Elsaygh et al., 2023). Specifically, the peaks at 3419.9 cm^−1^ (O–H/N–H stretching) and 1128.4 cm^−1^ (C–O stretching) indicated a polysaccharide backbone, whereas the twin peaks at 2924.3 and 2854.7 cm^−1^ (aliphatic C–H stretching) confirm the presence of hydrophobic fatty acid chains. Furthermore, the prominent amide I band at 1631.1 cm^−1^ (C=O stretching) revealed the presence of a glycoprotein complex structure, which is consistent with that of other extremotolerant bioemulsifiers (Adetunji and Olaniran, 2019; Rojas-Vargas et al., 2023). Such Emulsan polymers generally possess a high hydrophilic–lipophilic balance (HLB > 10) and resist denaturation across wide pH ranges (3–12) and high-salinity conditions (D’Almeida et al., 2024). By deploying this resilient Emulsan, strain A11 effectively disperses the hydrophobic pyrene, mitigating mass transfer limitations for the core-degrading strain N7.

The extremely low water solubility of HMW-PAHs greatly limits the rate of microbial degradation. Using the HADEG database (Dams-Kozlowska et al., 2008), no relevant bioemulsifier-producing genes were annotated in the genome of strain N7. In the genome of strain A11, *wza*, *wzb*, and *wzc* related genes were identified (Table 2), which were established as core indicator genes of emulsan (Nakar and Gutnick, 2001). Moreover, genes encoding lipopolysaccharide biosynthesis protein, O-antigen polymerase, nonhydrolyzing UDP-N-acetylglucosamine 2-epimerase, etc., and bioemulsifiers synthesizing and secreting genes were also annotated in strain A11, which were similar to alleles in *Acinetobacter* sp. RAG-1 (Cébron et al., 2008). The result indicating that strain A11 may able to synthesis lipopolysaccharide-like bioemulsifier in genome aspect.

**TABLE 2:** Functional genes related to bioemulsifier synthesis and export in strain A11.

Functional module	Gene	Accession number	A11 BLAST Result (nr) (Description / Accession / Identity)
Export system	*wza (weeK)*	FPDNJMPM_01277	Polysaccharide biosynthesis/export family protein (WP_004882133.1 / 100.00%)
	*wzb*	FPDNJMPM_01276	Low molecular weight protein-tyrosine-phosphatase (WP_004882134.1 / 100.00%)
	*wzc*	FPDNJMPM_01275	Polysaccharide biosynthesis tyrosine autokinase (WP_004882135.1 / 100.00%)
Transmembrane flippase	*wzx*	FPDNJMPM_01274	Lipopolysaccharide biosynthesis protein (WP_257736273.1 / 99.50%)
Chain polymerization	*wzy*	FPDNJMPM_01301	O-antigen polymerase (WP_004882124.1 / 100.00%)
Core modification	*weeB (ugd)*	FPDNJMPM_01300	UDP-N-acetyl-D-mannosamine dehydrogenase (WP_004882130.1 / 100.00%)
Lipid modification	*weeJ (acylT)*	FPDNJMPM_01296	DegT/DnrJ/EryC1/StrS family aminotransferase (WP_004882107.1 / 100.00%)
Backbone assembly	*weeG*	FPDNJMPM_01306	Glycosyltransferase family 4 protein (WP_004882113.1 / 100.00%)
Precursor modification	*weeA*	FPDNJMPM_01299	non-hydrolyzing UDP-N-acetylglucosamine 2-epimerase (WP_004882132.1 / 100.00%)
Precursor supply	*galU*	FPDNJMPM_01143	UTP-glucose-1-phosphate uridylyltransferase GalU (WP_004882103.1 / 100.00%)
	*galE*	FPDNJMPM_00824	UDP-glucose 4-epimerase GalE (WP_004882096.1 / 100.00%)
Chaperone protein	*mip*	FPDNJMPM_03717	FKBP-type peptidyl-prolyl cis-trans isomerase (WP_019385335.1 / 100.00%)

### 3.6 Functional gene expression by the consortium PES during pyrene degradation

**FIGURE 7:**
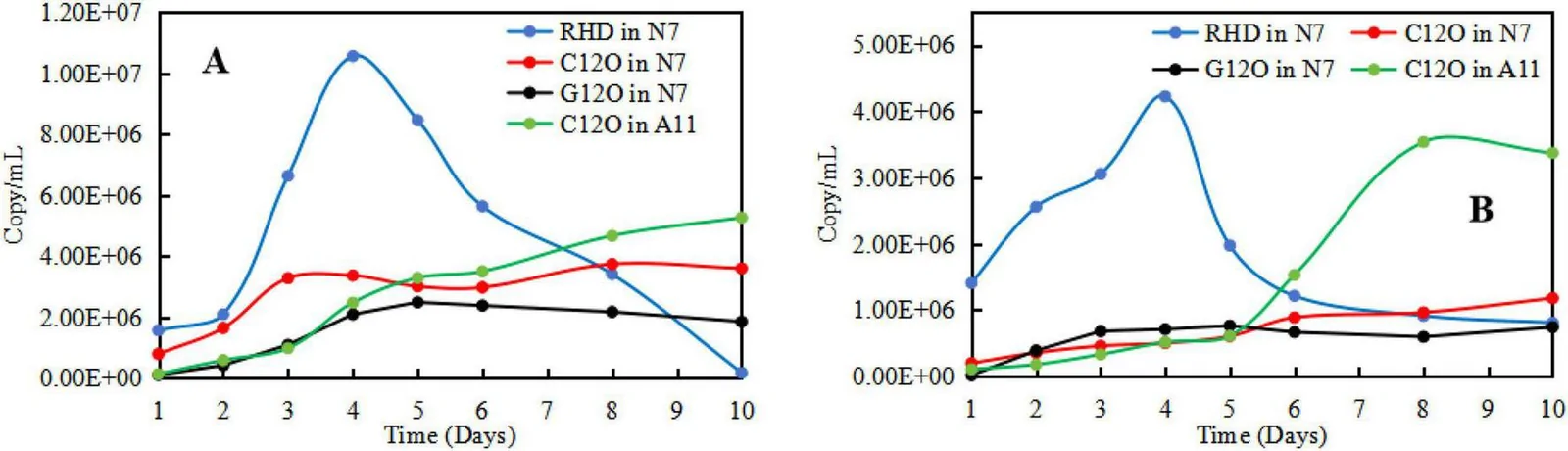
Expression of functional genes in pyrene-degrading process by consortium PES, **(A)** at 5% salinity, **(B)** at 10% salinity.

Through an in-depth study of the genomic information of strain A11, several genes related to PAH degradation were identified (Supplementary Table 2). The presence of genes encoding salicylate hydroxylase and C12O indicated that strain A11 is able to degrade salicylic acid through the C12O pathway, which is an important PAH downstream degradation pathway (Wang et al., 2018). PAH-degrading gene clusters were also identified in the genome of strain N7 as described by Li et al. (2024), which contained genes encoding RHD, G12O and C12O. To identify the expression of important functional genes involved in pyrene degradation by the consortium PES, q-PCR experiments targeting genes encoding RHDs, G12O, and C12O in strain N7 and genes encoding C12O in strain A11 were performed in the consortium PES at 5% salinity and 10% salinity. As shown in Figure 7A. All functional genes were strongly expressed during pyrene degradation at both 5% and 10% salinity. Specifically, under the 5% salinity condition, the expression level of the RHD-encoding gene in strain N7 peaked at the early stage of degradation (day 4), reaching a maximum value of 1.05 × 10^7^ copies/mL. The rapid response of initial oxidation genes and subsequent decline have been reported in earlier dynamic studies (Paissé et al., 2012), possibly because of the prevention of redundant metabolic consumption (Liang et al., 2019). During the subsequent degradation phase, both the C12O and G12O genes maintained consistently prominent expression levels. Notably, the expression level of the C12O gene in strain A11 was greater than that of the downstream degradation genes in strain N7, which demonstrated that strain A11 played a critical role in the transformation of downstream intermediate metabolites during the degradation process. In contrast, under 10% salinity conditions, the expression levels of these genes significantly decreased. The maximum value of the RHD-encoding gene reached only 4.22 × 10^6^ copies/mL. The expression levels of the other genes also decreased markedly compared with those under 5% salinity. However, the expression of the gene encoding C12O in strain A11 was relatively high compared with that in the other strains. However, compared with that of other downstream degradation genes, the relative expression level of the C12O-encoding gene in strain A11 significantly increased under 10% salinity. These findings indicated that strain A11 played a more critical role in the downstream degradation of pyrene under high-salt conditions and elucidated the reason why the environmental tolerance of the synthetic microbial consortium was superior to that of pure cultures at the functional gene expression level.

### 3.7 Syntrophic network of pyrene degradation by the consortium PES

**FIGURE 8:**
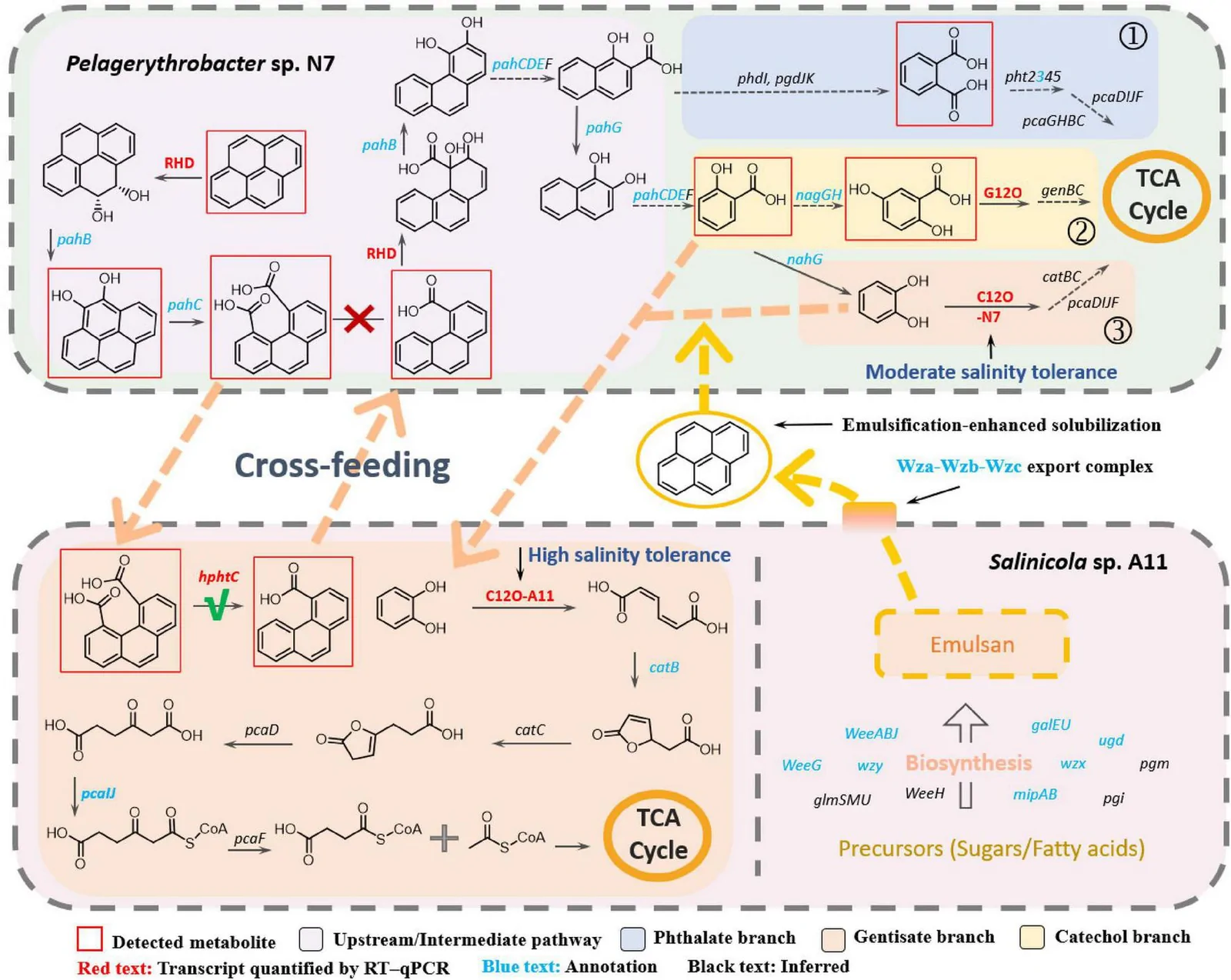
The proposed syntrophic network of pyrene degradation by consortium PES.

The results of intermediate detection, genome annotation, and bioemulsifier synthesis and gene expression analyses revealed a syntrophic network of pyrene degradation by consortium PES was proposed (Figure 8). Pyrene was initially transformed into 4,5-dihydroxy-4,5-dihydropyrene under the action of RHD encoded by the pahA gene in strain N7 and was subsequently converted to 4,5-dihydroxypyrene and 4,5-dicarboxyphenanthrene by the enzymes encoded by pahB and pahC. However, strain N7 lacked the capacity to further transfer 4,5-dicarboxyphenanthrene. Under the catalysis of the decarboxylase encoded by the hpthC-5 gene in strain A11, 4,5-dicarboxyphenanthrene was further converted to 4-phenanthroic acid for subsequent degradation.

The metabolic flux subsequently entered the phenanthrene degradation pathway. Under the catalysis of enzymes encoded by pahA and pahB in strain N7, 4-phenanthroic acid was converted to 1-hydroxy-2-naphthoic acid, and complete mineralization occurred via the G12O and C12O pathways. Strain N7 could carry out subsequent downstream degradation through both the C12O and G12O pathways, while strain A11 may participated in the downstream degradation of pyrene exclusively via the C12O pathway. Moreover, the bioemulsifiers secreted by strain A11 were exported to the extracellular environment via the Wza-Wzb-Wzc transmembrane system, which significantly increased the hydrophilicity and bioavailability of pyrene and other related hydrophobic organic pollutants. To our knowledge, this is the first report of a syntrophic degradation network of pyrene by halophiles, and the first time 4,5-dicarboxyphenanthrene was proposed as the key intermediate in the HMW-PAH syntrophic degradation process.

## 4 Conclusions

In this study, a halophilic synthetic pyrene-degrading consortium, PES, consisting of *Pelagerythrobacter* sp. N7 and *Salinicola* sp. A11, was constructed using a bottom-up approach. Consortium PES achieved complete degradation of 50 mg/L pyrene within 10 days at 5% salinity and exhibited good environmental tolerance. On the basis of the detection of intermediate metabolites, genomic analysis, expression of key functional genes, and bioemulsifier characterization, we proposed a syntrophic network model of pyrene degradation by consortium PES. Strain N7 was responsible for the main upstream degradation process of pyrene but failed to complete the full mineralization of pyrene because of the deficiency of 4,5-dicarboxyphenanthrene decarboxylase. Strain A11 had no direct pyrene degradation capacity but could assist in degradation by providing 4,5-dicarboxyphenanthrene decarboxylase and participating in the downstream degradation of pyrene via the C12O pathway. Moreover, strain A11 could synthesize and secrete bioemulsifiers, which increased the bioavailability of pyrene and other related hydrophobic organic pollutants, thereby further improving the pyrene degradation efficiency of the consortium.

## Data Availability

The datasets presented in this study can be found in online repositories. The names of the repository/repositories and accession number(s) can be found in the article/Supplementary material.

## References

[B1] Adetunji AI Olaniran AO. (2019). Production and characterization of bioemulsifiers from Acinetobacter strains isolated from lipid-rich wastewater. *Biotech*. 9:151. 10.1007/s13205-019-1683-y 30944798 PMC6434010

[B2] Al-Thukair A. A. Malik K. Nzila A. (2020). Biodegradation of selected hydrocarbons by novel bacterial strains isolated from contaminated Arabian Gulf sediment. *Sci. Rep*. 10 21846. 10.1038/s41598-020-78733-0 33318512 PMC7736303

[B3] Badejo A. C. Badejo A. O. Shin K. H. Chai Y. G. (2013). A gene expression study of the activities of aromatic ring-cleavage dioxygenases in mycobacterium gilvum PYR-GCK to changes in salinity and pH during pyrene degradation. *PLoS One*. 8:e58066. 10.1371/journal.pone.0058066 23469141 PMC3585252

[B4] Beygisangchin M. Jakmunee J. Kamarudin S. K. Baghdadi A. H. Saetang S. Thipprasert S. (2026). A critical review of polycyclic aromatic hydrocarbons and their adverse effects on human health: Insights from the past six years. *Environ. Sci. Eur.* 38:87. 10.1186/s12302-026-01367-y

[B5] Budiyanto F. Thukair A. Al-Momani M. Musa M. M. Nzila A. (2018). Characterization of halophilic bacteria capable of efficiently biodegrading the high-molecular-weight polycyclic aromatic hydrocarbon pyrene. *Environ. Eng. Sci.* 35 616–626. 10.1089/ees.2017.0244

[B6] Capitani G. Tramonti A. Bossa F. Grütter M. G. De Biase D. (2003). The critical structural role of a highly conserved histidine residue in group II amino acid decarboxylases. *FEBS Lett*. 554 41–44. 10.1016/s0014-5793(03)01079-2 14596911

[B7] Cébron A. Norini M. P. Beguiristain T. Leyval C. (2008). Real-time PCR quantification of PAH-ring hydroxylating dioxygenase (PAH-RHDalpha) genes from gram positive and gram negative bacteria in soil and sediment samples. *J. Microbiol. Methods* 73 148–159. 10.1016/j.mimet.2008.01.009 18329116

[B8] Chamanrokh P. Assadi M. M. Amoabediny G. Rashedi H. (2010). Cleaning oil-contaminated vessel by emulsan producers (autochthonous bacteria). *J. Environ. Health Sci. Eng.* 7 209–222.

[B9] Chebbi A. Hentati D. Zaghden H. Baccar N. Rezgui F. Chalbi M. et al. (2017). Polycyclic aromatic hydrocarbon degradation and biosurfactant production by a newly isolated *Pseudomonas* sp. strain from used motor oil-contaminated soil. *Int. Biodeterior. Biodegrad.* 122 128–140. 10.1016/j.ibiod.2017.05.006

[B10] Dai C. Han Y. Duan Y. Lai X. Fu R. Liu S. (2022). Review on the contamination and remediation of polycyclic aromatic hydrocarbons (PAHs) in coastal soil and sediments. *Environ. Res*. 205:112423. 10.1016/j.envres.2021.112423 34838568

[B11] D’Almeida A. P. de Albuquerque T. L. Rocha M. V. P. (2024). Recent advances in Emulsan production, purification, and application: Exploring bioemulsifiers unique potentials. *Int J. Biol. Macromol*. 278 133672. 10.1016/j.ijbiomac.2024.133672 38971276

[B12] D’Almeida A. P. de Azevedo D. C. S. Melo V. M. M. de Albuquerque T. L. Rocha M. V. P. (2024). Bioemulsifier production by acinetobacter venetianus AMO1502: Potential for bioremediation and environmentally friendly applications. *Mar. Pollut. Bull*. 203:116436. 10.1016/j.marpolbul.2024.116436 38762935

[B13] Dams-Kozlowska H. Mercaldi M. P. Panilaitis B. J. Kaplan D. L. (2008). Modifications and applications of the acinetobacter venetianus RAG-1 exopolysaccharide, the emulsan complex and its components. *Appl. Microbiol. Biotechnol*. 81 201–210. 10.1007/s00253-008-1664-2 18795287

[B14] Dhote M Kumar A Juwarkar A. (2018). Petroleum contaminated oil sludge degradation by defined consortium: Influence of biosurfactant production. *Proc. Natl. Acad. Sci. India Sect. B Biol. Sci.* 88:517–523. 10.1007/s40011-016-0778-z

[B15] Duraimurugan R. Vignesh K. Sahasra C. Parthipan P. Narenkumar J. Rajasekar A. (2025). Characterization of halophilic bacteria *Vreelandella piezotolerans* DM1 on biodegradation of polyaromatic hydrocarbons and its pathway. *Water Air Soil Pollut.* 236:807. 10.1007/s11270-025-08449-2

[B16] Eaton R. W. (2001). Plasmid-encoded phthalate catabolic pathway in Arthrobacter keyseri 12B. *J. Bacteriol*. 183 3689–3703. 10.1128/JB.183.12.3689-3703.2001 11371533 PMC95246

[B17] Elsaygh Y. A. Gouda M. K. Elbahloul Y. Hakim M. A. El Halfawy N. M. (2023). Production and structural characterization of eco-friendly bioemulsifier SC04 from Saccharomyces cerevisiae strain MYN04 with potential applications. *Microb. Cell Fact*. 22 176. 10.1186/s12934-023-02186-z 37679768 PMC10485968

[B18] Embarcadero-Jiménez S. Araujo-Palomares C. L. Moreno-Perlín T. Ramírez-Álvarez N. Quezada-Hernández C. Batista-García R. A. (2024). Physiology and comparative genomics of the haloalkalitolerant and hydrocarbonoclastic marine strain rhodococcus ruber MSA14. *Arch. Microbiol*. 206:328. 10.1007/s00203-024-04050-z 38935150

[B19] Fan W. Jin J. Zhang Z. Han L. Li K. Wang C. (2022). Degradation of phenanthrene by consortium 5H under hypersaline conditions. *Environ. Pollut*. 308:119730. 10.1016/j.envpol.2022.119730 35809715

[B20] Ghorbannezhad H. Moghimi H. Dastgheib S. M. M. (2022). Biodegradation of high molecular weight hydrocarbons under saline condition by halotolerant Bacillus subtilis and its mixed cultures with *Pseudomonas* species. *Sci. Rep*. 12:13227. 10.1038/s41598-022-17001-9 35918482 PMC9345985

[B21] Jumper J. Evans R. Pritzel A. Green T. Figurnov M. Ronneberger O. et al. (2021). Highly accurate protein structure prediction with alphaFold. *Nature*. 596 583–589. 10.1038/s41586-021-03819-2 34265844 PMC8371605

[B22] Karmakar M. Jana D. Manna T. Mitra M. Guchhait K. C. Dey S. (2024). Bioremediation by Brevibacterium sediminis: a prospective pyrene degrading agent to eliminate environmental polycyclic aromatic hydrocarbons. *World J. Microbiol. Biotechnol*. 40:377. 10.1007/s11274-024-04178-6 39495360

[B23] Kim S. J. Kweon O. Jones R. C. Freeman J. P. Edmondson R. D. Cerniglia C. E. (2007). Complete and integrated pyrene degradation pathway in mycobacterium vanbaalenii PYR-1 based on systems biology. *J. Bacteriol*. 189 464–472. 10.1128/JB.01310-06 17085566 PMC1797382

[B24] Kumari B Rajput S Gaur P Singh SN Singh DP. (2014). Biodegradation of pyrene and phenanthrene by bacterial consortium and evaluation of role of surfactant. *Cell Mol. Biol.* 60:22–28. 10.14715/cmb/2014.60.5.5 25535708

[B25] Kweon O. Kim S. J. Holland R. D. Chen H. Kim D. W. Gao Y. (2011). Polycyclic aromatic hydrocarbon metabolic network in mycobacterium vanbaalenii PYR-1. *J. Bacteriol*. 193 4326–4337. 10.1128/JB.00215-11 21725022 PMC3165511

[B26] Li X. Cao X. Zhang Z. Li Y. Zhang Y. Wang C. et al. (2024). Mechanism of phenanthrene degradation by the halophilic Pelagerythrobacter sp. *N7 Chemosphere* 350:141175. 10.1016/j.chemosphere.2024.141175 38211788

[B27] Li Y. Li W. Ji L. Song F. Li T. Fu X. (2022). Effects of salinity on the biodegradation of polycyclic aromatic hydrocarbons in oilfield soils emphasizing degradation genes and soil enzymes. *Front. Microbiol*. 12:824319. 10.3389/fmicb.2021.824319 35087508 PMC8787140

[B28] Liang C Huang Y Wang H. (2019). PAHE, a functional marker gene for polycyclic aromatic hydrocarbon-degrading bacteria. *Appl. Environ. Microbiol*. 85 e02399-18. 10.1128/AEM.02399-18 30478232 PMC6344622

[B29] Liang Y. Gardner D. R. Miller C. D. Chen D. Anderson A. J. Weimer B. C. et al. (2006). Study of biochemical pathways and enzymes involved in pyrene degradation by Mycobacterium sp. strain KMS. *Appl. Environ. Microbiol*. 72 7821–7828. 10.1128/AEM.01274-06 17041157 PMC1694249

[B30] Lv M. Luan X. Liao C. Wang D. Liu D. Zhang G. et al. (2020). Human impacts on polycyclic aromatic hydrocarbon distribution in Chinese intertidal zones. *Nat. Sustain.* 3 878–884. 10.1038/s41893-020-0565-y

[B31] Naidoo G. Naidoo K. (2021). Salinity exacerbates oil contamination effects in mangroves. *Environ. Sci. Pollut. Res. Int*. 28 68398–68406. 10.1007/s11356-021-15450-9 34272666

[B32] Nakar D. Gutnick D. L. (2001). Analysis of the wee gene cluster responsible for the biosynthesis of the polymeric bioemulsifier from the oil-degrading strain acinetobacter lwoffii RAG-1. *Microbiology*. 147 1937–1946. 10.1099/00221287-147-7-1937 11429470

[B33] Nzila A. Ramirez C. O. Musa M. M. Sankara S. Basheer C. Li Q. X. (2018). Pyrene biodegradation and proteomic analysis in *Achromobacter xylosoxidans*. *Int. Biodeterior.* 130 40–47. 10.1016/j.ibiod.2018.03.014

[B34] Paissé S. Goñi-Urriza M. Stalder T. Budzinski H. Duran R. (2012). Ring-hydroxylating dioxygenase (RHD) expression in a microbial community during the early response to oil pollution. *FEMS Microbiol. Ecol*. 80 77–86. 10.1111/j.1574-6941.2011.01270.x 22136434

[B35] Qin R Xu T Jia X. (2022). Engineering *Pseudomonas* putida to produce rhamnolipid biosurfactants for promoting phenanthrene biodegradation by a two-species microbial consortium. *Microbiol. Spectr*. 10:e0091022. 10.1128/spectrum.00910-22 35730952 PMC9431653

[B36] Qutob M. Rafatullah M. Muhammad S. A. Alosaimi A. M. Alorfi H. S. Hussein M. A. (2022). A review of pyrene bioremediation using *Mycobacterium* strains in a different matrix. *Fermentation* 8:260. 10.3390/fermentation8060260

[B37] Rojas-Vargas J. Castelán-Sánchez H. G. Pardo-López L. (2023). HADEG: A curated hydrocarbon aerobic degradation enzymes and genes database. *Comput. Biol. Chem*. 107:107966. 10.1016/j.compbiolchem.2023.107966 37778093

[B38] Schöning-Stierand K. Diedrich K. Fährrolfes R. Flachsenberg F. Meyder A. Nittinger E. (2020). Proteinsplus: Interactive analysis of protein-ligand binding interfaces. *Nucleic Acids Res*. 48 W48–W53. 10.1093/nar/gkaa235 32297936 PMC7319454

[B39] Sun S. Wei R. Hu S. Yang M. Ni J. (2024). Isolation and characterization of distinctive pyrene-degrading bacteria from an uncontaminated soil. *Biodegradation*. 35 657–670. 10.1007/s10532-023-10065-y 38279065

[B40] Sun W. Yang H. Rao R. Zhang L. Ke C. Wang S. et al. (2025). *Bacillus* B3 degrades pyrene in oil-contaminated soil via a novel phthalate pathway and efficient bioremediation. *Environ. Technol. Innov.* 40:104509. 10.1016/j.eti.2025.104509

[B41] Trott O. Olson A. J. (2010). Autodock vina: Improving the speed and accuracy of docking with a new scoring function, efficient optimization, and multithreading. *J. Comput. Chem*. 31 455–461. 10.1002/jcc.21334 19499576 PMC3041641

[B42] Uzoigwe C. Burgess J. G. Ennis C. J. Rahman P. K. (2015). Bioemulsifiers are not biosurfactants and require different screening approaches. *Front. Microbiol*. 6 245. 10.3389/fmicb.2015.00245 25904897 PMC4387539

[B43] Vaidya S. Jain K. Madamwar D. (2017). Metabolism of pyrene through phthalic acid pathway by enriched bacterial consortium composed of *Pseudomonas*. *Biotech*. 7:29. 10.1007/s13205-017-0598-8 28401465 PMC5388654

[B44] Volkamer A. Kuhn D. Rippmann F. Rarey M. (2012). DoGSiteScorer: A web server for automatic binding site prediction, analysis and druggability assessment. *Bioinformatics* 28 2074–2075. 10.1093/bioinformatics/bts310 22628523

[B45] Wanapaisan P. Laothamteep N. Vejarano F. Chakraborty J. Shintani M. Muangchinda C. (2018). Synergistic degradation of pyrene by five culturable bacteria in a mangrove sediment-derived bacterial consortium. *J. Hazard Mater*. 342 561–570. 10.1016/j.jhazmat.2017.08.062 28886568

[B46] Wang C. Guo G. Huang Y. Hao H. Wang H. (2017). Salt adaptation and evolutionary implication of a nah-related PAHS dioxygenase cloned from a halophilic phenanthrene degrading consortium. *Sci. Rep*. 7:12525. 10.1038/s41598-017-12979-z 28970580 PMC5624874

[B47] Wang C. Huang Y. Zhang Z. Wang H. (2018). Salinity effect on the metabolic pathway and microbial function in phenanthrene degradation by a halophilic consortium. *AMB Express*. 8:67. 10.1186/s13568-018-0594-3 29696463 PMC5918149

[B48] Wang C. Huang Y. Zhang Z. Hao H. Wang H. (2020). Absence of the nahG-like gene caused the syntrophic interaction between marinobacter and other microbes in PAH-degrading process. *J. Hazard Mater*. 384:121387. 10.1016/j.jhazmat.2019.121387 31648897

[B49] Wang J. Chitsaz F. Derbyshire M. K. Gonzales N. R. Gwadz M. Lu S. (2023). The conserved domain database in 2023. *Nucleic. Acids Res*. 51 D384–D388. 10.1093/nar/gkac1096 36477806 PMC9825596

[B50] Wang W. Wang L. Shao Z. (2018). Polycyclic Aromatic Hydrocarbon (PAH) Degradation Pathways of the Obligate Marine PAH Degrader Cycloclasticus sp. *Strain P1. Appl Environ. Microbiol*. 84 e1261–e1218. 10.1128/AEM.01261-18 30171002 PMC6193391

[B51] Zhao B. Wang H. Mao X. Li R. (2009). Biodegradation of phenanthrene by a halophilic bacterial consortium under aerobic conditions. *Curr. Microbiol*. 58 205–210. 10.1007/s00284-008-9309-3 19011941

[B52] Zhou H. Wang H. Huang Y. Fang T. (2016). Characterization of pyrene degradation by halophilic *Thalassospira* sp. strain TSL5-1 isolated from the coastal soil of Yellow Sea. *China. Int. Biodeterior. Biodegrad.* 107 62–69. 10.1016/j.ibiod.2015.10.022

[B53] Zhuo Y. Jin C. Z. Lee C. S. Lee H. G. (2025). Functional and genomic insights into BHET-degrading *Stenotrophomonas* sp. isolated from the marine plastisphere. *Front. Microbiol*. 16:1680692. 10.3389/fmicb.2025.1680692 41209732 PMC12588907

