# Correction: The syntrophic network of pyrene degradation by the synthetic halophilic consortium PES

**DOI:** 10.3389/fmicb.2026.1971230

**Published:** 2026-09-03

**Authors:** Yilong Wen, Haoze Lu, Lin Wang, Han Zhao, Guang Guo, Fang Tian, Chongyang Wang, Shuxian Dang

**Affiliations:** 1 Miami College, Henan University, Kaifeng, Henan, China; 2 College of Environmental Engineering, Nanjing Institute of Technology, Nanjing, China

**Keywords:** pyrene, synthetic consortium, 4, 5-dicarboxyphenanthrene, bioemulsifier, syntrophic network

There was an error within Figure 8 as published. The chemical structures representing 4,5-dicarboxyphenanthrene and 4-carboxyphenanthrene were inadvertently interchanged. As a result, their positions in the HphtC-mediated decarboxylation step are reversed in the published Figure 8.

The correct sequence should be:

4,5-dicarboxyphenanthrene → 4-carboxyphenanthrene

The updated Figure 8 is below:

The original version of this article has been updated.

**Figure 8:**
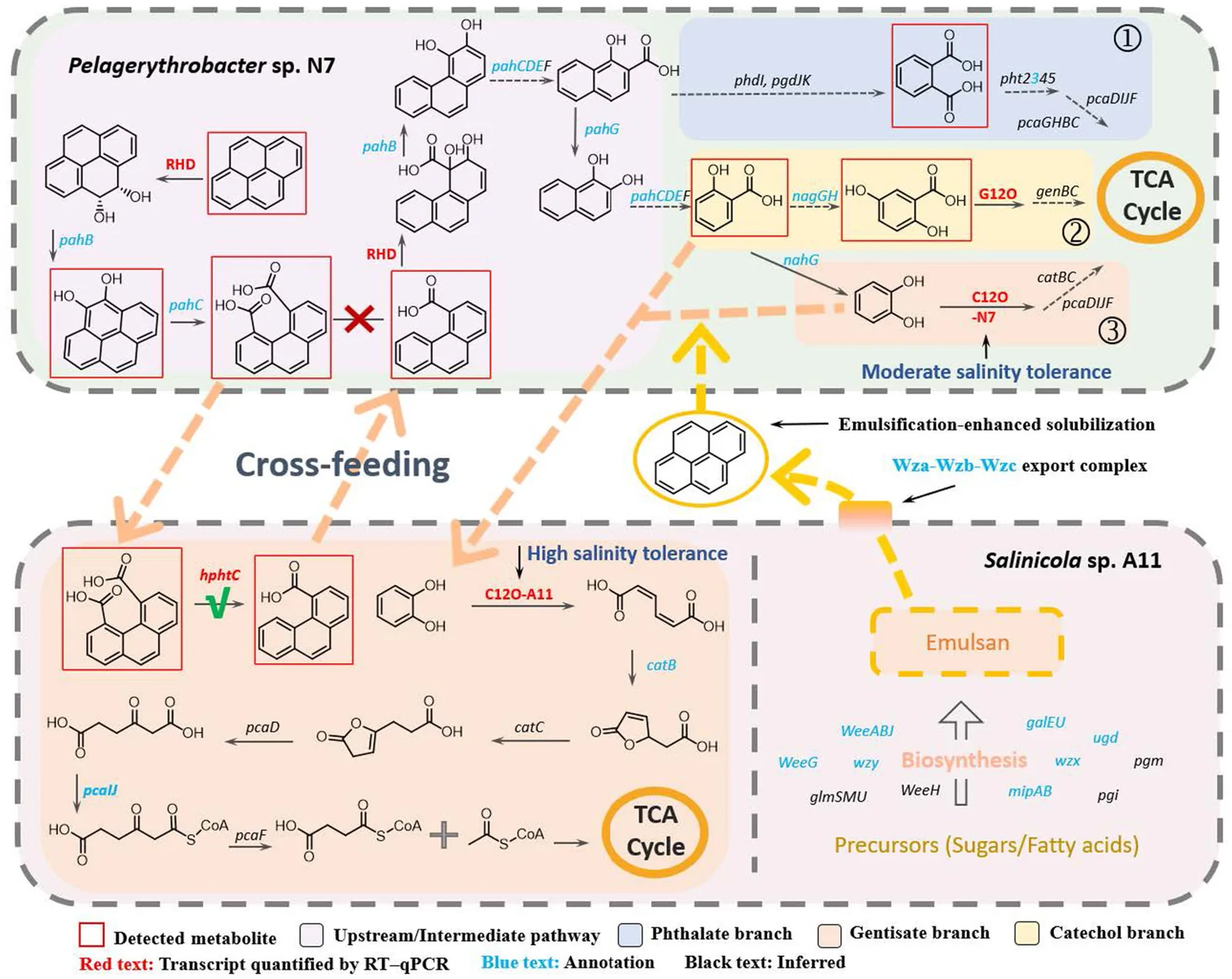
The proposed syntrophic network of pyrene degradation by consortium PES.

