# Supplementary material for: The syntrophic network of pyrene degradation by the synthetic halophilic consortium PES
Source: Front Microbiol. 2026 Aug 14;17:1899654. doi: 10.3389/fmicb.2026.1899654 (PMC13522185; doi:10.3389/fmicb.2026.1899654)
Supplement: Supplementary file 1 [file Table_1.docx]

**Table S1 primers of q-PCR used in this study**

| No. | Primers 5’-3’ | Target genes | Annealing temperature | Length of the fragment |
| --- | --- | --- | --- | --- |
| 1 | GACTATCTGGGCGATGTCCG | RHD | 60 | 160 |
|  | CATGAGTCCACCCAACGTGA |  |  |  |
| 2 | GAAAGAGGTCGTGCGGGAAT | C12O | 54 | 271 |
|  | GCGTCCCGACAGATGAAGAA |  |  |  |
| 3 | GTCTCTATGCGGGCCTTCAA | G12O | 59 | 232 |
|  | GGAATATCGAGCCCGTCGAG |  |  |  |
| 4 | GCGAAGTCGTCGAAGGTGTA | C12O-A11 | 55 | 305 |
|  | GCACCCTCTATACCGACAGC |  |  |  |
| 5 | TCTTCGAGACACGAAACCGC | hphtC | 52 | 230 |
|  | GGCAAGGAGCACTTCCTGAT |  |  |  |

**Table S2 PAH-degrading related genes contained in strain A11**

| **KO** | **Gene** | **Definition** | **EC** |
| --- | --- | --- | --- |
| K01857 | *pcaB* | 3-carboxy-cis,cis-muconate cycloisomerase | 5.5.1.2 |
| K10218 | *ligK* | 4-hydroxy-4-methyl-2-oxoglutarate aldolase | 4.1.3.17 |
| K14727 | *pcaL* | 4-carboxymuconolactone decarboxylase | 4.1.1.44 |
| K01826 | *hpaF* | 5-carboxymethyl-2-hydroxymuconate isomerase | 5.3.3.10 |
| K01821 | *praC* | 4-oxalocrotonate tautomerase | 5.3.2.6 |
| K00483 | *hpaB* | 4-hydroxyphenylacetate 3-monooxygenase | 1.14.14.9 |
| K14519 | *aldH* | NADP-dependent aldehyde dehydrogenase | 1.2.1.4 |
| K00529 | *hcaD* | 3-phenylpropionate/trans-cinnamate dioxygenase ferredoxin reductase component | 1.18.1.3 |
| K00480 | *nahG* | salicylate hydroxylase | 1.14.13.1 |
| K14519 | *aldH* | NADP-dependent aldehyde dehydrogenase | 1.2.1.4 |
| K03381 | *catA* | Catechol 1,2-dioxygenase | 1.13.11.1 |
| K01856 | *catB* | muconate cycloisomerase | 5.5.1.1 |
| K01031 | *pcaI* | 3-oxoadipate CoA-transferase subunit A | 2.8.3.6 |
| K01032 | *pcaJ* | 3-oxoadipate CoA-transferase subunit B | 2.8.3.6 |

Fig. S1 the standard curve of different functional genes obtained in RT-PCR detection. **A** gene encoding RHD in strain N7, **B** gene encoding G12O in strain N7, **C** gene encoding C12O in strain N7, **D** gene encoding C12O in strain A11, **E** hphtC-5 in strain A11.
